# Reactive oxygen species induce Cox-2 expression via TAK1 activation in synovial fibroblast cells

**DOI:** 10.1016/j.fob.2015.06.001

**Published:** 2015-06-06

**Authors:** Yuta Onodera, Takeshi Teramura, Toshiyuki Takehara, Kanae Shigi, Kanji Fukuda

**Affiliations:** Division of Cell Biology for Regenerative Medicine, Institute of Advanced Clinical Medicine, Kindai University Faculty of Medicine, Osaka, Japan

**Keywords:** COX, cyclooxygenase, HA, hyaluronic acid, NAC, N-acetyl cysteamine, OA, osteoarthritis, PGs, prostaglandins, RA, rheumatoid arthritis, ROS, reactive oxygen species, SFs, synovial fibroblast cells, Reactive oxygen species, Cox-2, TAK1, Synovial tissues, OA model

## Abstract

Oxidative stress within the arthritis joint has been indicated to be involved in generating mediators for tissue degeneration and inflammation. COX-2 is a mediator in inflammatory action, pain and some catabolic reactions in inflamed tissues. Here, we demonstrated a direct relationship between oxidative stress and Cox-2 expression in the bovine synovial fibroblasts. Furthermore, we elucidated a novel mechanism, in which oxidative stress induced phosphorylation of MAPKs and NF-κB through TAK1 activation and resulted in increased Cox-2 and prostaglandin E2 expression. Finally, we demonstrated that ROS-induced Cox-2 expression was inhibited by supplementation of an antioxidant such as N-acetyl cysteamine and hyaluronic acid *in vitro* and *in vivo*. From these results, we conclude that oxidative stress is an important factor for generation of Cox-2 in synovial fibroblasts and thus its neutralization may be an effective strategy in palliative therapy for chronic joint diseases.

## Highlights

• Oxidative stress in the arthritis joint is involved in generating mediators for inflammation.
• Oxidative stress-induced expression of Cox-2 was mediated by MAPKs and NF-κB.
• ROS-induced MAPKs and NF-κB were attenuated by inhibition of MAPKKK TAK1.
• Inhibition of TAK1 activity resulted in reduced expression of Cox-2 and PGE2.
• ROS-induced TAK1 activation and Cox-2 expression was inhibited by antioxidants N-acetyl cysteamine and hyaluronic acid.

## 1 Introduction

Reactive oxygen species (ROS) produced by normal metabolism are important biological mediators in the cellular signaling cascades [1]. Appropriate amounts of ROS have beneficial effects on several physiological processes including protection from pathogens, wound healing, and tissue regeneration [2,3]. On the other hand, harmful levels of ROS could be generated in response to ultraviolet radiation [4], cigarette smoking [5], alcohol [6], some drugs [7,8], ischemia–reperfusion injury [9] and inflammatory disorders [10]. Disproportionate levels of ROS give rise to further disease development such as cardiovascular disease, neurodegeneration, cancer and chronic inflammation [3,5].

Oxidative stresses in the joint have been indicated as being involved as inflammatory mediators in the arthritis [2,11]. Intra-articular ROS may be responsible for collagen hydrolysis and activation of metalloproteinase, leading to the degradation of the extracellular matrix in cartilage [12–15], and resulting in the pathogenesis of osteoarthritis (OA) [11,16,17]. Rheumatoid arthritis (RA), which is an autoimmune inflammatory disease, is also accompanied by oxidative stress that can directly contribute toward the destructive, proliferative synovitis evident in RA [2,18].

In the chronic arthritis such as RA or OA, high levels of cyclooxygenase (COX)-2 are detected [19,20]. There are two isoforms of the COX enzymes: COX-1 is expressed constitutively in most cells. COX-2, on the other hand, is dramatically up-regulated by inflammation and contributes to producing prostaglandins (PGs), which mediate a number of the characteristic features of inflammation and reactions leading to the tissue damage [21]. In the arthritis, localization of Cox-2 in vascular endothelial cells, infiltrating mononuclear inflammatory cells, and subsynovial fibroblast-like cells are well observed [19]. Some researchers have reported that inflammatory cytokines, such as interleukin (IL)-1 and tumor necrosis factor (TNF)-α, and growth factors, such as platelet-derived growth factor (PDGF), positively regulate COX-2 expression. [22,23].

Interestingly, the relationship between ROS and Cox-2 has been reported in the different cell types. Barbieri et al. elucidated that Cox-2 expression could be triggered by ROS through activation of NF-κB and ERK1/2 in macrophage [24]. Mari et al. demonstrated that supplementation of antioxidants diminished IL-1α induced Cox-2 expression in intestinal myofibroblasts [22]. Unfavorable ROS and/or Cox-2 are clearly important therapeutic targets for chronic joint inflammation, thus elucidation of the relationship between ROS and Cox-2 would be an important factor in developing effective treatments.

The present study was designed to (i) evaluate the profile of Cox-2 expression after ROS addition *in vitro*, and (ii) understand the mechanisms of how ROS enhance Cox-2 expression in synovial fibroblasts.

## 2 Materials and methods

### 2.1 Animal use and care

The use of animals complied with the regulations of the Institutional Animal Use and Care Committee of the Kinki University Faculty of Medicine. Each mouse was housed in an individual pen with food and water ad libitum. Animals were exposed to an artificially controlled light–dark regime with 14 h (hours) lighting and 10 h darkness. Temperature was maintained between 20 °C and 25 °C in a ventilated room. All operations were performed under anesthesia induced by abdominal cavity injection of 50 mg/kg sodium pentobarbital and local injection of 2% Xylocaine with epinephrine.

### 2.2 Culture of the synovial fibroblast cells (SFs)

Synovial tissues were collected from the metacarpophalangeal (MP) joints of freshly slaughtered calves about 10 months of age, which were donated from a local slaughterhouse. The SFs were isolated from the synovial tissues by enzymatic digestion with 2 mg/ml of collagenase (Wako Pure Chemical Industries, Osaka, Japan) for 6 h at 37 °C. After filtration, cells were seeded in culture plates and cultured in 10% FCS supplemented alpha-MEM at 37 °C and 5% CO_2_, 20% O_2_ for 48 h.

### 2.3 Reagents treatment

Prior to exposure of H_2_O_2_, the SFs were pre-cultured in serum-free culture medium composed of serum-free culture medium consisting of 0.1% bovine serum albumin (BSA, Sigma–Aldrich, St. Louis, MO, USA), alpha-MEM (Life technologies Inc., Carlsbad, CA, USA), 1% insulin-transferrin selenium (Life technologies) and 1% antibiotic/antimycotic solution (Life Technologies) for 24 h. The SFs were cultured under these conditions for 24 h when the following reagents were added; 100 μM H_2_O_2_ (Wako Pure Chemical Industries), 10 μM p38MAPK specific inhibitor SB203580 (Wako), 5 μM MEK/Erk specific inhibitor PD0325901 (Wako), 10 μM JNK specific inhibitor SB600125 (Wako), 10 μM NF-κB specific inhibitor Bay 11-7082 (Sigma), 5 μM TAK1 specific inhibitor 5Z-7-Oxozeaenol (Sigma), 2 mg/ml hyaluronic acid (HA) (SUVENYL, approx. 1900 kDa, CHUGAI PHARMACEUTICAL CO., LTD., Tokyo, Japan), 100 μM N-Acetylcysteine (Nacalai teque, Kyoto, Japan).

### 2.4 Measurement of intracellular reactive oxygen species (ROS)

Generation of ROS was detected using 3′-p-(aminophenyl) fluorescein (APF) (Sekisui Medical CO. LTD., Tokyo, Japan) *in vitro*. Bovine SFs at passage 2 were seeded on 6-well plate, and incubated with 5 μM APF (final concentration) for 30 min at 37 °C. After washing twice with culture medium, the cells were treated with H_2_O_2_ with/without HA for 2 h. Then, the cells were dispersed by TrypLE Express (Life Technologies), re-suspended with PBS and put into 96-well black bottom assay plates (Corning, NY, USA). Fluorescence intensities were analyzed by a Wallac ARVO MX 1420 multilabel counter (Perkin Elmer Japan, Kanagawa, Japan).

### 2.5 Quantitative RT-PCR (qRT-PCR) analysis

Total RNA was extracted with the TRIzol reagent (Invitrogen) and reverse transcribed with the High Capacity cDNA reverse transcription kit (Applied Biosystems, Foster City, CA, USA). The qRT-PCR with total cDNA was performed using Perfect real-time SYBR green II (Takara Bio, Inc., Shiga, Japan) with specific primers (Table 1) in the Thermal Cycler Dice® Real Time System (Takara Bio, Inc.) at 95 °C for 20 s followed by 40 cycles of 95 °C for 5 s, 60 °C for 30 s. To quantify the relative expression of each gene, the Ct (threshold cycle) values were normalized to an endogenous reference (ΔCt = Ct_target_ − Ct_Gapdh_) and compared with a calibrator (control), using the ΔΔCt method (ΔΔCt = ΔCt_sample_ − ΔCt_calibrator_).

### 2.6 Western blot (WB) analysis

The SFs were lysed with the LIPA buffer and mixed with SDS buffer (4% SDS, 125 mM Tris–HCl, 10% 2-mercaptoethanol, 0.01% bromophenol blue in 20% glycerol), then the total cell lysates were separated by SDS–PAGE and transferred to polyvinylidene difluoride (PVDF) membranes. (Hybond-P; Amersham Pharmacia Biotech, Buckinghamshire, UK). The blotted membranes were blocked at room temperature for 1 h with Block Ace (Dainippon Pharmaceutical, Osaka, Japan) and treated with the following primary antibodies (Table 2) overnight at 4 °C. Detection was realized by enhanced chemiluminescence with an ImmunoStar® LD (Wako) and horseradish peroxidase (HRP)-conjugated secondary antibodies (all were purchased from Santa Cruz Biotechnology, CA, USA) corresponding to each primary antibody. The lumino-labeled membranes were analyzed by the Amersham™ Imager 600 (GE Healthcare, Tokyo, Japan).

### 2.7 Enzyme-linked immunosorbent assay (ELISA)

To estimate the PGE2 concentrations in the SFs, the samples were lysed in 15% methanol in 0.1 M sodium phosphate buffer (pH 7.5), centrifuged at 12,000 rpm for 5 min and then the supernatant was collected. For each assay, 100 μl of the supernatant of the lysates was added to a well of the 96-well ELISA plate from the Human PGE2 ELISA kit (OXFORD BIOMEDICAL RESERCH, MI, USA) and sequentially treated with detection antibodies and chromogenic substrate according to the manufacturer’s instructions. The reaction was terminated by adding 50 μl of 1 M H_2_SO_4_ to the well, and the optical density was read at 450 nm. The PGE2 concentration was determined based on a standard curve that was produced using the serially diluted reference samples provided in the PGE2 ELISA kit.

### 2.8 Surgical OA model mice and drug treatment

Experimental OA was induced by surgical destabilization of the medial meniscus (DMM), as described previously [25,26]. Only 8-weeks (wks) old female mice were used in our study. Under anesthesia, the right knee joint capsule was exposed and the medial meniscotibial ligament was transected under microscope to give destabilization of the medial meniscus. A sham operation was performed on the left knee joint in which the ligament was visualized but not transected. Four weeks after the surgery, they received intra-articular injection of 20 μl of HA (SUVENYL, CHUGAI PHARMACEUTICAL CO., LTD.) or the same volume of saline as control. Four days after treatments, they were sacrificed and used for the analysis.

### 2.9 Histology and fluorescence microscopy

To observe the ROS accumulation, we used the Protein Carbonyls Immunohistochemical Staining Kit (SHIMA Laboratories, Co., LTD., Tokyo, Japan). Generated ROS in the cells or tissues react with circumjacent protein and modified lysine, arginine, proline, and threonine side chain amines into aminoacyl carbonyls. Thus we can detect and quantify the accumulation of ROS as the existence of the carbonylated protein [27]. Immunofluorescence and histological analysis were performed according to the manufacture’s instruction of a Protein Carbonyls Immunohistochemical Staining Kit. The knee joints were fixed and decalcified with decalcifying liquid K-CX (FALMA, Tokyo, Japan). For histological observation, the samples were dehydrated and embedded in paraffin. The sections were then stained with Safranin-O (Sigma) or double-stained with Alcian blue (Wako). The knee joints were deparaffinized and rehydrated paraffin sections were blocked with Block Ace for 1 h, then washed twice with 0.1% Triton-TBS (TBS-T), and incubated with 1/300 diluted each antibody (Table 3) at 4 °C overnight. The specimens were then washed twice with TBS-T containing 10% Block Ace and incubated with 1/1000 diluted FITC-conjugated anti-rabbit IgG bovine secondary antibody. After two washes, 1/1000 diluted DAPI and observed using a fluorescence microscope (BZ-9000, Keyence corporation, Osaka, Japan). 2,4-Dinitrophenol (DNP) and Cox-2 positive cells in the cartilage-synovial junction were quantified by BZ-9000 software (Keyence corporation).

### 2.10 Statistical analysis

Significant difference was detected by Tukey–Kramer HSD test or Student’s *t*-test. A *p*-value of less than 0.05 was considered significant.

## 3 Results

### 3.1 ROS induced expression of Cox-2 and PGE2 in the SFs

**Fig. 1:**
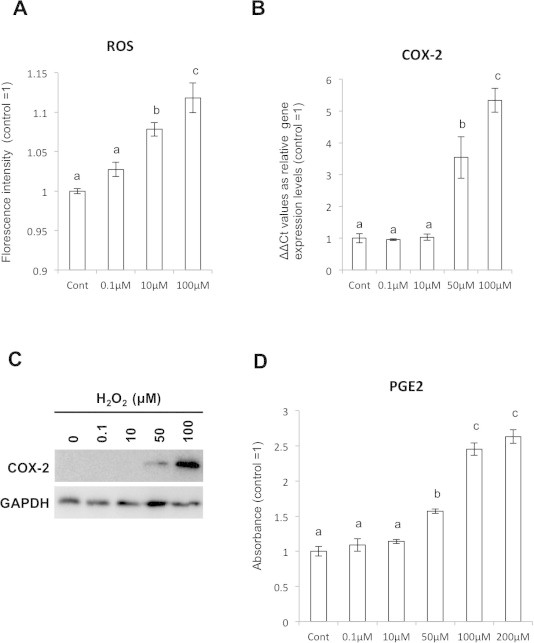
H_2_O_2_ addition induced Cox-2 expression in SFs. (A) Estimation of intracellular ROS after H_2_O_2_ stimulation to the SFs by APF staining. (B) qPCR assay for Cox-2 mRNA expression after H_2_O_2_ treatment. (C) Cox-2 protein expression in the H_2_O_2_-treated SFs shown by WB. (D) Increasing of PGE2 expression by H_2_O_2_ stimulation in dose dependent manner was detected by ELISA assay.

We first determined if the ROS induced Cox-2 generation in the SFs. H_2_O_2_ clearly increased intracellular ROS levels in a dose dependent manner (Fig. 1A). Similarly, qRT-PCR revealed that the Cox-2 mRNA expression increased depending on the concentration of H_2_O_2_ (Fig. 1B). To confirm the expression of Cox-2 in protein levels, we performed WB assay and observed significant increase of Cox-2 proteins when the cells were stimulated by H_2_O_2_ at 50 μM and 100 μM (Fig. 1C). We then performed ELISA assay for PGE2 downstream molecule of Cox-2. The results were consistent with ELISA, in which significant up-regulation of PGE2 (Fig. 1D).

### 3.2 MAPKs and NF-κB activities potentially mediated ROS induced Cox-2 and PGE2 up-regulation

**Fig. 2:**
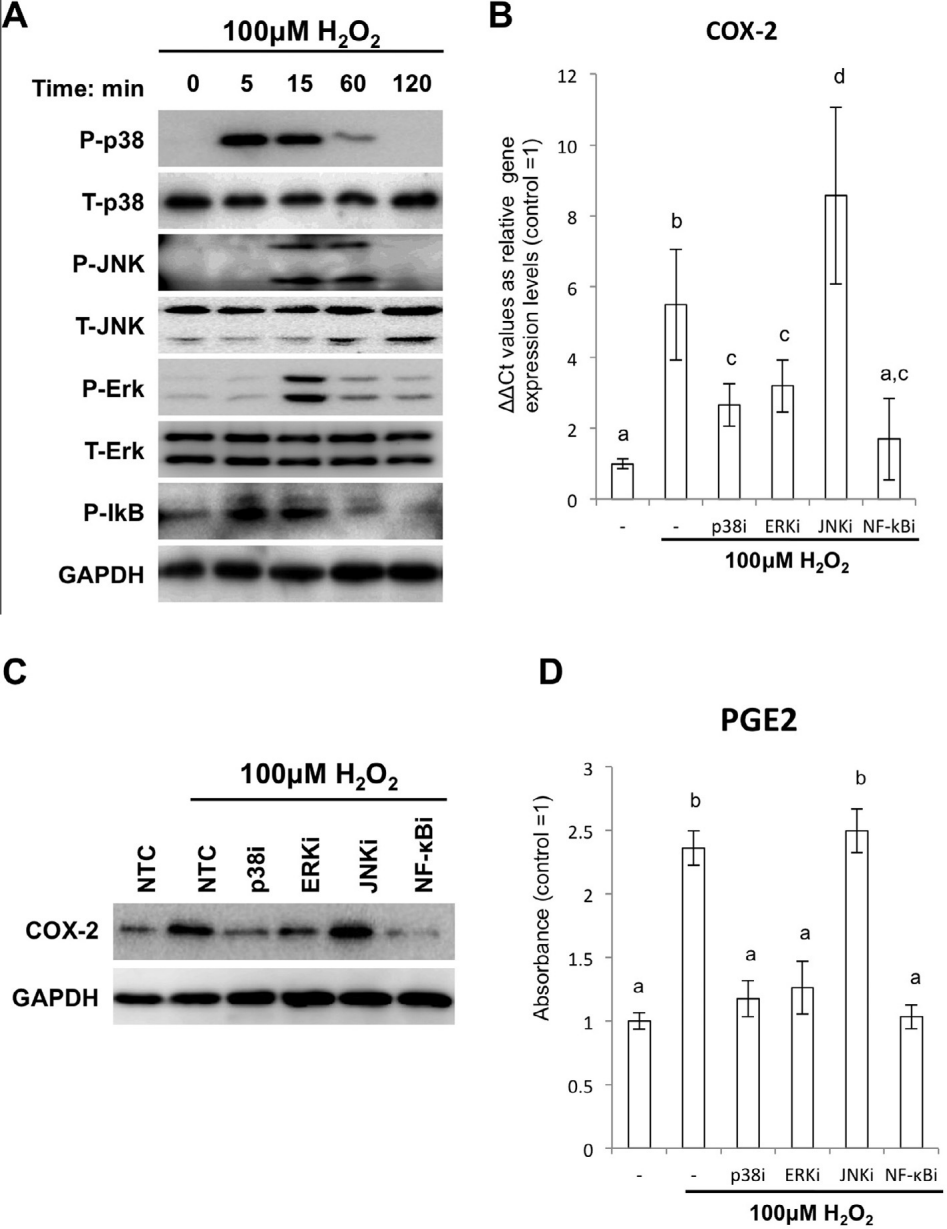
H_2_O_2_ addition induced Cox-2 and PGE2 expression through phosphorylation of MAPKs and IkB in the SFs. (A) Detection of phosphorylated p38, JNK, Erk and IκB in the H_2_O_2_-treated SFs by WB. (B) qPCR assay for the Cox-2 expression in the SFs treated with H_2_O_2_ and MAPKs or NF-κB inhibitors. *Y*-axis shows relative expression values to the untreated control. (C) WB analysis for Cox-2 expressions in the SFs treated with H_2_O_2_ and MAPKs or NF-κB inhibitors. (D) ELISA assays for PGE2 expression in the SFs treated with H_2_O_2_ and MAPKs or NF-κB inhibitors. *Y*-axis shows staining intensity (absorbance) of each sample.

Previous studies demonstrated that MAPKs and/or NF-κB could be the second messenger in the ROS-induced signaling pathways in macrophage [24]. Thus we hypothesized that the MAPKs and/or NF-κB play an important role for the ROS- induced Cox-2 expression also in the SFs. After stimulation of the SFs by 100 μM of H_2_O_2_, clear enhancement in phosphorylated p38, JNK, Erk and IκB, which is a marker for NF-κB activity, were detected by WB analysis (Fig. 2A). To confirm if the phosphorylated status of MAPKs and NF-κB relates to Cox-2 and subsequent PGE2 expressions, we treated the SFs by H_2_O_2_ with MAPKs or NF-κB inhibitors followed by examined Cox-2 and PGE2 expressions. When the SFs were treated by H_2_O_2_ in the presence of p38 inhibitors, H_2_O_2_-enhanced Cox-2 expression was abolished. Similarly, ERK or NF-κB inhibitors also significantly attenuated H_2_O_2_-enhanced Cox-2 expression, respectively. On the other hand, JNK inhibitor did not show any effect on the ROS-induced Cox-2 expression (Fig. 2B). These results were verified by WB analysis: Cox-2 protein accumulation by H_2_O_2_ was attenuated by treatment with p38, ERK or NF-κB inhibitors (Fig. 2C). Then we confirmed that the alteration in the Cox-2 expression was reflected into the downstream PGE2 by ELISA assay. The ELISA assay clearly showed similar trend with that of Cox-2: H_2_O_2_ addition induced about 2.2 times increase in the PGE2 protein, and p38, ERK and NF-κB inhibitors attenuated the expression levels to the baseline control, respectively (Fig. 2D).

### 3.3 TAK1 mediated H_2_O_2_-indiced MAPKs and NF-κB activations

**Fig. 3:**
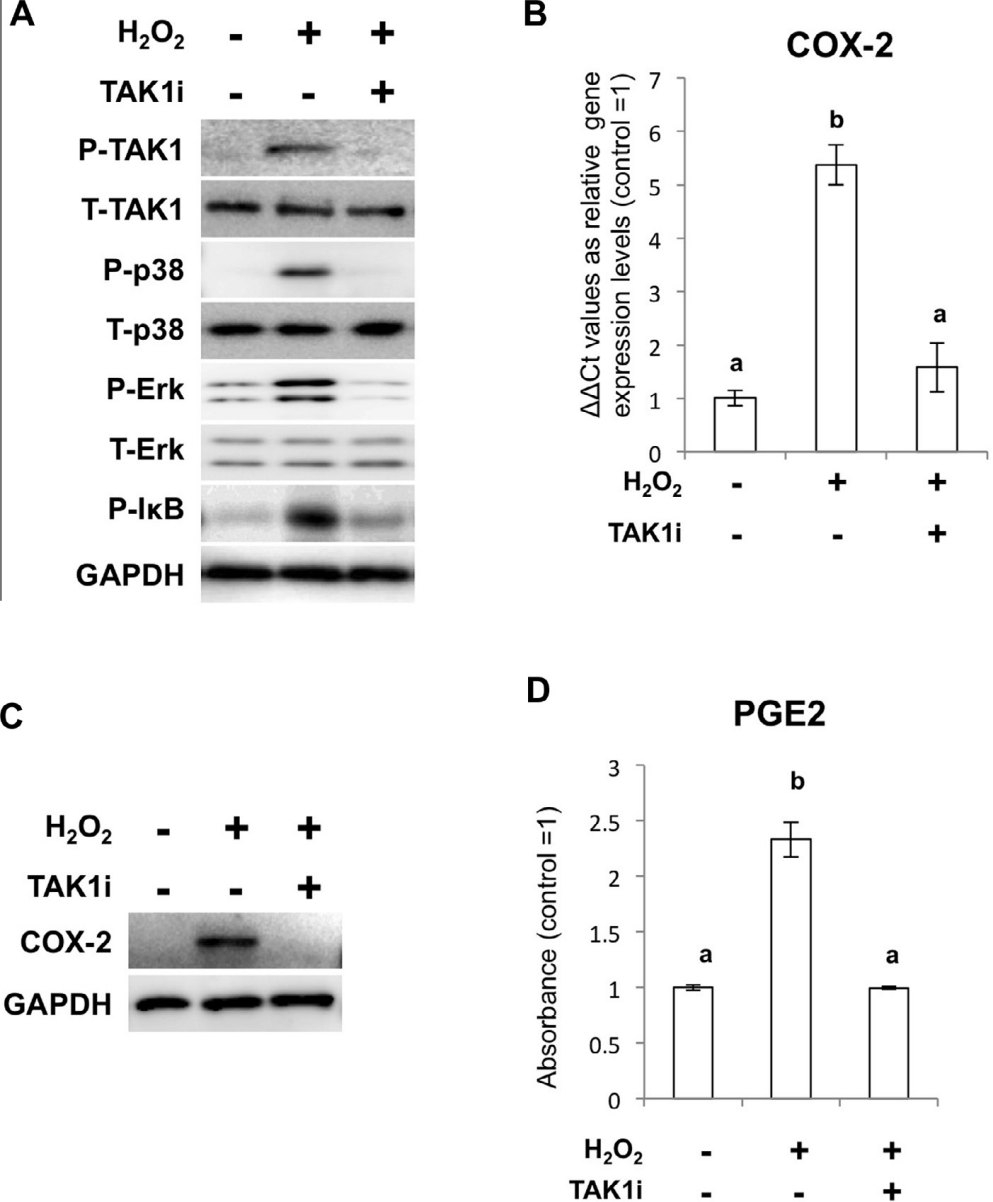
TAK1 is involved in H_2_O_2_ mediated activation of MAPKs and NF-κB signaling cascades. (A) WB analysis for the phosphorylated status of MAPKs and IκB in the cells treated with H_2_O_2_ and TAK1 inhibitor. (B) pPCR analysis for Cox-2 expression in the cells treated with H_2_O_2_ and TAK1 inhibitor. (C) WB analysis for Cox-2 expression after H_2_O_2_ stimulation and TAK1 inhibition. (D) ELISA analysis for PGE2 expression after H_2_O_2_ stimulation and TAK1 inhibition.

Since multiple MAPKs and NF-κB were simultaneously activated by ROS, we hypothesized that some upstream molecules were responsible for the ROS-induced up-regulation of Cox-2 and PGE2 expressions. Accumulating evidence suggests the involvement of TAK1 in the ROS-induced MAPKs activation [28,29]. Thus we looked at the effect of H_2_O_2_ in the phosphorylation of TAK1 and found that H_2_O_2_ clearly caused the phosphorylation of TAK1 in the SFs. Supplementation of TAK1 inhibitor blocked phosphorylation of the downstream molecules p38, Erk and NF-κB, respectively (Fig. 3A). Importantly, Inhibition of TAK1 during H_2_O_2_ treatment blocked both the expression of Cox-2 and PGE2 completely on RNA (Fig. 3B) and protein levels (Fig. 3C and D). This action was conserved in other cell types (Supplement 1).

### 3.4 Supplementation of N-acetyl cysteamine (NAC) and hyaluronic acid (HA) resulted in repression of Cox-2 and PGE2 expressions

**Fig. 4:**
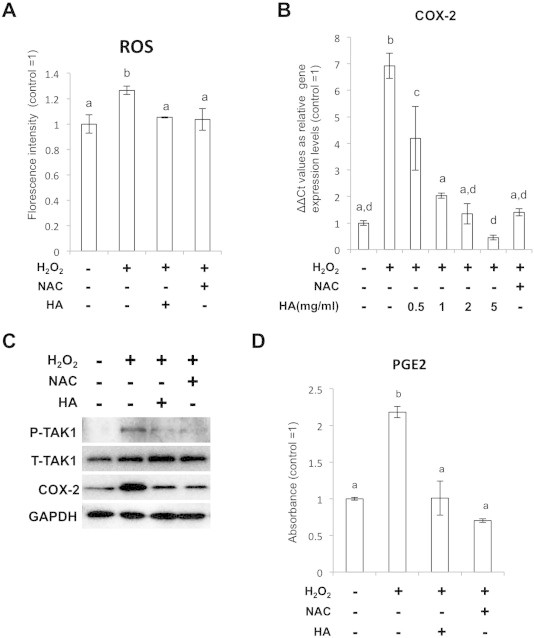
Antioxidants attenuate ROS-mediated Cox2 and PGE2 expressions. (A) Estimation of the intracellular ROS levels in the cells treated with 100 μM H_2_O_2_ and 100 μM NAC or 2 mg/ml HA. (B) qPCR analysis for Cox-2 expression in the SFs treated with H_2_O_2_ and 100 μM NAC or HA at 0.5, 1, 2 and 5 mg/ml. (C) WB showing suppression of TAK1 phosphorylation and Cox2 expression in the cells treated with NAC and HA. (D) ELISA assays for PGE2 expression in the SFs treated with 100 μM H_2_O_2_, 100 μM NAC and 2 mg/ml HA.

As the last part of the study, we examined if the supplementation of antioxidant could suppress the ROS-induced Cox-2 expression both *in vitro* and *in vivo*. When we treated the SFs with H_2_O_2_ and NAC, which is a potent ROS scavenger due to its cysteine residues, accumulation of intracellular ROS was blocked (Fig. 4A). HA is also a potent ROS scavengers [30,31]. Importantly, phosphorylation of TAK1 was inhibited in both NAC and HA-added conditions (Fig. 4B) and Cox-2 expression was repressed to the similar level with that of the baseline control by treatment with the antioxidants (Fig. 4C). Then we observed PGE2 expression in H_2_O_2_ and HA or NAC treated cells by ELISA. The ELISA assay clearly showed that the expression of PGE2 was also significantly attenuated in HA or NAC-treated cells (Fig. 4D). Finally, we tried to observe the relationship between TAK1, Cox-2 and HA in the surgical OA models, which has significant synovial expansion and inflammation.

**Fig. 5:**
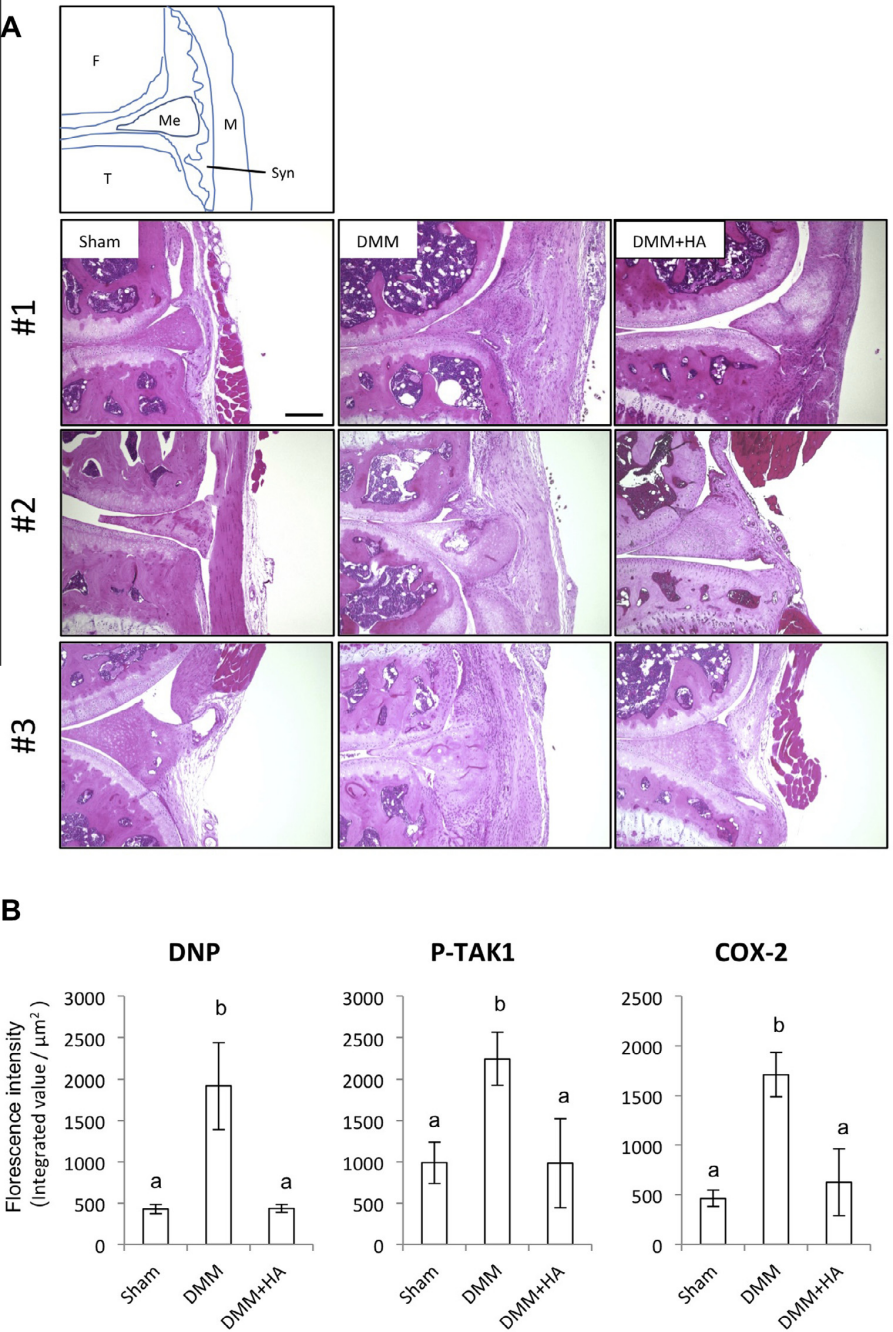
Intra-articular injection of HA attenuated ROS accumulation and Cox-2 expression in the surgical OA mice. (A) Triplicate sections with hematoxylin and eosin (HE) staining from sham, DMM, and DMM joint treated with HA. F: Femur, T: Tibia, Me: Meniscus, Syn: Synovia, M: Muscle. Scale bar = 200 μm. (B) Quantified expression of immunofluorescence for the DNP, phosphorylated TAK1 and Cox-2. Different characters mean significant differences between each group.

In the joint of the DMM-induced OA model mice, clear synovial thickening was observed at 4 wks after surgery (Fig. 5A). In the lesions, strong DNP signals reflecting accumulation of the ROS-dependent carbonylated protein was detected. Furthermore, expression of phosphorylated TAK1 and Cox-2 were observed. In the OA mice injected with HA, ROS-derived protein carbonylation, detected with the DNP expression, was attenuated (Supplement Fig. 3) although thickness of the synovial tissue was not affected (data not shown). Both phosphorylated TAK1 and Cox-2 expression were also completely inhibited (Fig. 5B).

## 4 Discussion

Here we described the relationship between ROS and Cox-2 expression in the SFs and explored a mechanism: ROS activates TAK1-MAPKs/NF-κB, which is an upstream molecular pathway for Cox-2 expression and PGE2 production.

In the present study we first demonstrated that the ROS induced Cox-2 expression in the SFs. Some previous studies have shown that Cox-2 expression is regulated by MAPKs (p38, ERK, etc) and NF-κB [32–34]. These MAPKs or NF-κB control the Cox-2 expression through directly binding to the promoter region of Cox-2 or via regulation of other transcription factors [35–38]. Furthermore the activated MAPKs and NF-κB can induce production of inflammatory cytokines [39,40] that strongly induce various catabolic factors. In the present study, we demonstrated that the activations of the p38, ERK and NF-κB directly link to the expression level of Cox-2, consistent with the previous report [41]. On the other hand, we observed that the inhibition of JNK activity, in turn, further enhanced H_2_O_2_-induced Cox-2 expression. In general, the function of JNK is closely linked with the Cox-2 generation, but JNK does not contribute to the stress-induced Cox-2 expression cascades [38,42,43], probably it depends on the cell and stress types loaded on the cells. We also confirmed that ROS stimulation induced phosphorylation of MAPKs and NF-κB in the SFs. Until now, there have been some papers suggesting the relationship between ROS accumulation and MAPK activation. In terms of the chondrocytes, the enhanced p38 phosphorylation with ROS has been reported [44]. Phosphorylations of ERK, JNK and NF-κB by ROS also have been described in various cell types [34,45,46]. These studies support the validity of our observation that ROS activated MAPKs and NF-κB in the SFs, and provide evidence that both MAPKs and NF-κB activations have important roles for the ROS-induced Cox-2 expression.

Furthermore, we hypothesized that ROS targets a common molecule located at upstream of both MAPKs and NF-κB. In this study, we focused on a candidate molecule TAK1, which could be a common upstream regulator of MAPKs and NF-κB [47–49]. Sato et al. demonstrated that IL-1β and TNFα caused phosphorylation of IκB and subsequent NF-κB activation, phosphorylation and activation of JNK and p38, leading to the impairment in embryonic fibroblast cells of TAK1 KO mouse [48]. This evidence clearly suggests that TAK1 is a critical regulator of both MAPKs and NF-κB. We actually demonstrated here that ROS induced TAK1 phosphorylation and subsequent Cox-2 expression. Furthermore, we observed that inhibition of TAK1 phosphorylation attenuated MAPK and NF-kB activation and also resulted in complete suppression of Cox-2 and PGE2 expressions. In terms of TAK1 induction by intracellular ROS generation, Wang et al. demonstrated in a pancreatic cancer cell with an anticancer drug Belinostat [29]. They observed that ROS generated by Belinostat induced TAK1 activation and resulted in AMPK-induced cell death. Chen et al. also reported that TAK1 activation occurred in H_2_O_2_ treated cardiomyocytes [28]. We demonstrated here that TAK1 was a primary effector molecule for ROS in normal cells, and the pathway from TAK1 activation to Cox-2 expression through MAPKs/NF-κB activations were significant target pathway for treatments of ROS-induced inflammation and pain. Contrarily, Hammaker et al. reported that decreasing the TAK1 expression did not affect the phosphorylation of p38 in the IL-1β treated synovial cells [50]. TAK1 binds to TAK1 binding proteins and other cofactors, and form TAK-1 complex to transduce stress signaling to MAPKs [51,52]. Thus it is possible that cofactors contribute to the variation of the reaction against IL-1 and ROS, although its precise role has to be elucidated.

Since ROS was a direct causative agent for Cox-2 expression and subsequent PGE2 production in the SFs, we then tested supplementation of ROS scavengers and examined if removing ROS can quench down the action of TAK1-MAPKs/Nf-kB-Cox2 and PGE2 cascades. We used two anti-ROS agent in this study; NAC, which is a by-product of glutathione and is popular as a potent scavenger of ROS due to its cysteine residues. HA, which is also a potential redox therapeutic molecule in the arthritic joint [30]. Underlying antioxidant mechanism of HA is due to chelation potentials of transition metals ions like Cu^2+^ or Fe^2+^, in which they lead to radical generation [31]. Addition of both NAC and HA attenuated ROS induced TAK1 phosphorylation and subsequent Cox-2 and PGE2 expressions in the SFs. These results may be an independent evidence for the idea that ROS directly induce TAK1 activation, MAPKs/NF-κB phosphorylation and subsequent Cox-2 and PGE2 expression. Finally, we performed intra-articular HA injection into the DMM model mice, and examined if the neutralization of OA-derived ROS can attenuate Cox-2 expression *in vivo*. As a result, HA injection into the joint clearly suppressed not only ROS accumulation but also Cox-2 generation. This result was consistent with the *in vitro* data, in which HA can suppress Cox-2 expression [53]. On the other hand, there are some papers showing that addition of HA resulted in up-regulation of Cox-2 in cancer cells or fibroblast cells [54–56]. One of the possible explanation is the phenotypic differences in the used cells, since HA plays a pivotal biological effect depending on the molecular weight [57]. Actually, inhibitory redox and Cox-2 effect was reported using high-molecular weight HA (HMW-HA) [58,59]. Furthermore, it has also been well known that p38 is phosphorylated by ROS and HMW-HA can attenuate its effect [60–62]. Contrarily, low-molecular weight HA (LMW-HA) induces MAPKs phosphorylation and subsequent Cox-2 expression [63,64]. Although further research will be necessary to elucidate the mechanisms, HMW-HA used in this study clearly suppressed ROS-induced p38 activation and Cox-2 expression.

Clinically, Cox-2 is believed to be a crucial therapeutic target because COX-2 contributes to the induction of PGE2 and other catabolic factors [20,65,66]. However, numerous studies suggest the negative side effect of the Cox2 inhibitors. On the other hand, development of antioxidant to reduce the ROS accumulations, which could be an essential factor for some chronic situations, has been an attractive research field. Actually, recent research suggested some significance of antioxidant in the treatment of arthritis.

In conclusion, we demonstrated a functional link between ROS and TAK1-MAPKs/NF-κB-Cox2-PGE2. This mechanism could be considered as a strategy to prevent arthritis.

## Author contributions

Y.O. and T.T. wrote the manuscript. T.T. and K.F. designed the study and experiments. Y.O., K.S. and T.T. performed the experiments.

## Conflict of interest statement

K.F. received a research grant from Chugai Pharmaceutical Co., Ltd.

## Figures and Tables

**Supplement 1:**
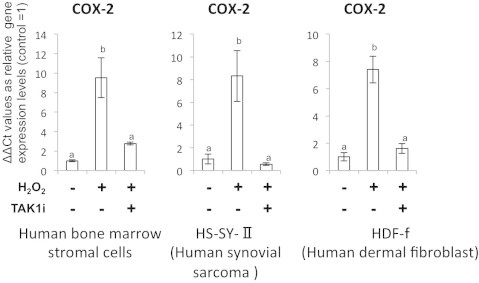


**Table 1:** Primer sequences used for qRT-PCR in the present study.

Primer name	Primer sequence (5′–3′)
Cox-2	F: ACAACAGAGTGTGTGATGTGC
	R: TGCTGTACGTAGTCTTCAATCAC

Gapdh	F: GTGAAGGTCGGAGTGAACG
	R: TAAAAGCAGCCCTGGTGAC

**Table 2:** Primary antibodies used in the WB experiments.

Antibody	Company	Dilution	Specific band (kDa)
Phospho p38 (Thr180/Tyr182, #4511)	Cell Signaling Technology	1/3000 in Immuno-enhancer	38
p38 (#9212)	Cell Signaling Technology	1/5000 in Immuno-enhancer	38
Phospho Erk1/2 (Thr202/Tyr204, #9101)	Cell Signaling Technology	1/3000 in Immuno-enhancer	42/44
Erk1/2 (#4695)	Cell Signaling Technology	1/5000 in Immuno-enhancer	42/44
Phospho JNK1/2 (Thr183/Tyr185, #4668)	Cell Signaling Technology	1/1000 in Immuno-enhancer	46/54
JNK1/2 (#9252)	Cell Signaling Technology	1/1000 in Immuno-enhancer	46/54
COX-2 (#12282)	Cell Signaling Technology	1/5000 in Immuno-enhancer	74
Phospho TAK1 (Thr184/187, #4531)	Cell Signaling Technology	1/1000 in Immuno-enhancer	82
TAK1 (#4505)	Cell Signaling Technology	1/1000 in Immuno-enhancer	82
I-kappa B alpha (sc-371)	Santa Cruz Biotechnology	1/1000 in Immuno-enhancer	35–41
Gapdh (3C2)	Abnova	1/10,000 in Immuno-enhancer	38

**Table 3:** Primary antibodies used in the immunohistochemistry.

Antibody	Company	Dilution
DNP(ROIK04)	SHIMA Laboratories	1/300 in 10% Block Ace/TBS-T
COX-2(#12282)	Cell Signaling Technology	1/300 in 10% Block Ace/TBS-T
Phospho TAK1(#4531)	Cell Signaling Technology	1/300 in 10% Block Ace/TBS-T
